# Epidemiologic study on changes in occurrence of hemorrhagic fever with renal syndrome in Republic of Korea for 17 years according to age group: 2001–2017

**DOI:** 10.1186/s12879-019-3794-9

**Published:** 2019-02-13

**Authors:** Yohan Park

**Affiliations:** Department of Internal Medicine, Yangju-si Public Health center, 1533 Buheung-ro, Yangju-si 11498, Gyeonggi-do, Republic of Korea

**Keywords:** Hemorrhagic fever with renal syndrome, Epidemiology, Age distribution, Seasonal variation, Immunization programs, Republic of Korea

## Abstract

**Background:**

The potential effect of the inactivated hantavirus vaccine (IHV) remains controversial; however, it appears to be moderately effective for patients at high risk of hemorrhagic fever with renal syndrome (HFRS). This study of the epidemiology of HFRS from 2001 to 2017 aimed to examine those at high risk of HFRS in the Republic of Korea (ROK), particularly in terms of disease distribution according to age.

**Methods:**

Raw data of HFRS patients recorded in Korea from 2001 to 2017 were obtained from the Korean Center for Disease Control and Prevention. Patients were divided into three age groups: ≤39, 40–69, and ≥ 70 years. The incidence rate per 100,000 individuals in each age group was calculated using population data. The 12-month year was divided into three-month quarters, and the number and proportion of patients corresponding to each quarter were calculated. The effects of time, sex, and quarter on HFRS incidence were assessed in a Poisson regression analysis.

**Results:**

From 2001 to 2017, 7048 HFRS patients were recorded nationwide. Among these patients, the proportion of patients aged ≥70 years increased gradually from 16.4% in 2001 to 43.9% in 2017. Regarding the quarter-year periods, the fourth quarter contained a significantly higher proportion of patients in the ≥70 years group (69.4%) compared to the other age groups. In the Poisson regression analysis, patients aged ≥70 years had a significantly higher relative risk of HFRS incidence within each quartile compared to those in the other age groups (2.102- and 10.029-fold in the third and fourth quarters, respectively). An analysis of disease incidence revealed a more distinct pattern in seasonal variation among those aged ≥70 years compared with other age groups.

**Conclusions:**

In this study of the incidence of HFRS in the ROK, subjects aged ≥70 years exhibited a gradual increase in incidence and a distinct pattern of seasonal variation. These results may be important to identify individuals in Korea who are at high risk of developing HFRS. In future, active immunization programs will be needed to control HFRS among these high-risk groups in Korea.

## Background

Hemorrhagic fever with renal syndrome (HFRS) is a serious infectious zoonotic disease that occurs in East Asia, including China and the Republic of Korea (ROK) [1–3]. HFRS is caused by hantaviruses; in the ROK, the *Hantaan* virus (HTNV) and *Seoul* virus (SEOV) are the primary etiological agents. HTNV infection usually occurs in rural areas, is associated with relatively severe symptoms, and varies seasonally, whereas SEOV infection tends to occur in urban areas, causes relatively mild symptoms, and does not vary seasonally [4, 5]. These viruses initially cause chronic infections in certain species of rodents and subsequently infect humans through aerosols and fomites from rodent feces, urine, and saliva [6–8]. Notably, the seasonal variations in HTNV infection, which usually affects people with primarily outdoor occupations, can be attributed to increased activities in rural areas, such as harvesting, and increased rodent activities, both of which occur in autumn [9, 10].

A previous epidemiologic study of HFRS in the ROK from 2001 to 2010 observed an incidence rate of 0.81 per 100,000 individuals; in that study, 82.1% of the patients were older than 40 years, and the case fatality rate was 1.01% [11]. According to a report from the Yearbook of Incidence for National Notifiable Infectious Diseases by the Ministry of Health and Welfare, ROK, the incidence of HFRS from 2011 to 2014 ranged from 0.7 to 1.0 per 100,000 individuals [12]. Furthermore, in an age decile-stratified analysis of data from 2001 to 2014 according to a report from the Korean Center for Disease Control and Prevention (KCDC), the highest number of cases were observed among those aged > 70 years (1395; 25.2%) [13].

A study of the incidence of HFRS in Shaanxi province, an endemic region in China, from 2005 to 2016 revealed an increase in the proportion of patients aged > 60 years over time (from 11.97% in 2005 to 25.45% in 2016) [14]. Another Chinese study conducted from 2005 to 2010 also reported a gradual increase in the incidence of HFRS in this population over time (from 8.8% in 2005 to 14.7% in 2010). The authors suggested two explanations for this phenomenon: an increase in the average age of the rural population and the influence of the Expanded Program on Immunization (EPI), which targets HFRS in endemic regions of China, on individuals aged 16–60 years [15].

The inactivated hantavirus vaccine (IHV) was developed in 1988 and is produced commercially under the name Hantavax®; it has been licensed for use in the ROK from 1990 [16, 17]. Immunogenicity data have led to recommendations to administer Hantavax® twice at a one-month interval, followed by a booster 12 months later [17–19]. To date, however, all IHV-related studies have had small samples and retrospective designs; in other words, the effectiveness of this vaccine has not been proven in well-designed clinical studies [20, 21]. Therefore, in the ROK, the population targeted by hantavirus immunization efforts is limited to people with high-risk outdoor occupations (e.g., soldiers or farmers), laboratory workers who handle HFRS-related viruses or experimental mice, and individuals considered at high risk of personal exposure. As the standard for inclusion in the target population is ambiguous, an active immunization program has not yet been conducted [22]. However, recent real-world data from China reported significant effects of the EPI, which targets HFRS [15, 23, 24].

Although HFRS is a rare disease, it is very dangerous and associated with a case fatality rate of 1%. Accordingly, a proper immunization program is needed in the ROK, and changes in the HFRS epidemiology should be investigated to provide a basis for selecting the target population. Therefore, this study investigated the epidemiology of HFRS in the ROK during a 17-year period (2001 to 2017) as well as the changes in overall and age-stratified patterns of HFRS incidence during this period.

## Methods

### Data collection

In the ROK, HFRS is classified as a national notifiable infectious disease (Category III); in other words, cases of this disease must be declared to the national government. Therefore, all HFRS patients can be identified from the National Notifiable Disease Surveillance System (NNDSS) website maintained by the KCDC. Using this resource, raw data regarding the numbers of HFRS patients by age, sex, month, and year and the incidence rate per 100,000 individuals in the ROK from 2001 to 2017 were obtained. The annual case fatality rate was calculated from the HFRS-related mortality data, which were available from 2011 to 2016. In addition, the total population stratified by year, age, and sex was obtained using population data provided by the Korea National Statistical Office.

To investigate the pattern of HFRS incidence according to age, patients were divided into three age groups: ≤39, 40–69, and ≥ 70 years. The 12-month year was divided into four quarters (January–March, April–June, July–September, and October–December). For each age group, the number and proportion of patients were investigated according to sex, year, and quarter, and trends in HFRS incidence within each age group were examined over time. Furthermore, the effects of time, sex, and quarter on HFRS incidence were analyzed overall and per age group. The incidence of HFRS per 100,000 individuals was calculated based on the annual population data, and the pattern of seasonal variation pattern in each age group was examined.

### Statistical analysis

Statistical analyses were performed using IBM® SPSS® software, version 18 (IBM Corporation, Armonk, NY, USA). The statistical significances of the numbers and proportions of patients in age-stratified groups were calculated using the χ^2^ test or Fisher’s exact test, appropriately. A Poisson generalized linear multiple regression analysis was performed to calculate the relative risks (RRs) of time, sex, and quarter with regard to the incidence of HFRS within each age group. *P*-values < 0.05 were considered to indicate statistical significance.

### Ethical review

This study was reviewed by the Institutional Review Board of the Korea National Institute for Bioethics Policy and it was confirmed that the utilization of the disease surveillance data did not require oversight by an ethics committee (P01–201808–21-003).

## Results

From 2001 to 2017, the cases of 7048 patients with HFRS were recorded in the ROK, yielding an average annual incidence of 0.83 per 100,000 residents. From 2011 to 2016, 31 patients died from HFRS, yielding a mean case fatality rate of 1.26%. Overall, 58.9% of patients were male, and 17.9, 54.1, and 28.0% had ages of ≤39, 40–69, and ≥ 70 years, respectively. The corresponding proportions of males in these age groups were 83.8, 55.9, and 48.8%, respectively, and this proportion was significantly higher among those aged ≤39 years (Table 1).

**Table 1:** Total number of patients, incidence rate, case fatality rate, and other aspects of HFRS

	Number of patients (%)		*P*-value^b^
Total number of patients (n)	7048		
Incidence rate (per 100,000 individuals) (mean ± standard deviation)	0.83 ± 0.14		
Total number of death (n)^a^	31		
Case fatality rate (%)^a^	1.26 ± 0.65		
Sex of patients			
Male (n, %)	4154 (58.9)		
Female (n, %)	2894 (41.1)		
Age of patients			
≤ 39 (n, %)	1261 (17.9)		
40~69 (n, %)	3813 (54.1)		
≥ 70 (n, %)	1974 (28.0)		
Proportion of sex by age group			
	Male	Female	
≤ 39 (n, %)	1057 (83.8)	204 (16.2)	< 0.001
40~69 (n, %)	2133 (55.9)	1680 (44.1)	< 0.001
≥ 70 (n, %)	964 (48.8)	1010 (51.2)	< 0.001

^a^Data for number of death were collected from 2011 to 2016

^b^Statistical significance was tested by χ^2^ test or Fisher’s exact test, appropriately

**Fig. 1:**
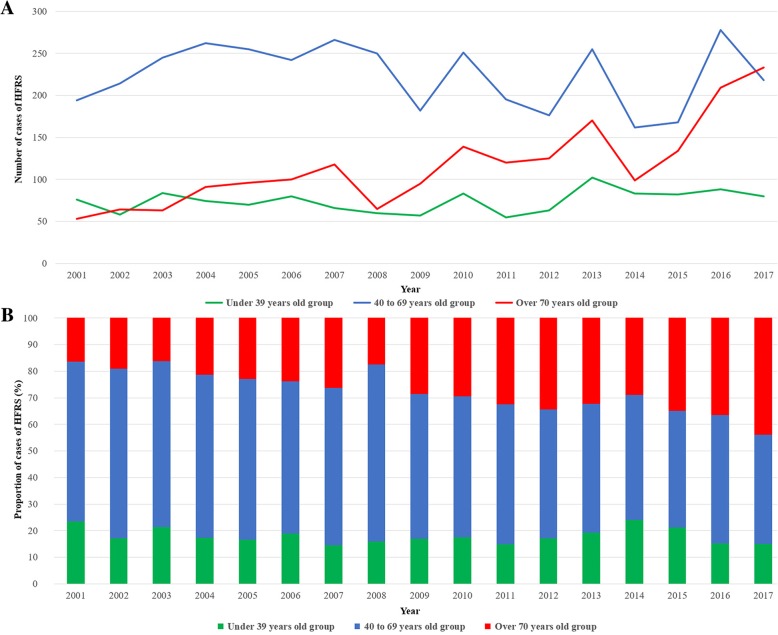
Changes in the number and proportion of HFRS patients over time in each age group (**a**) the changes of the number of HFRS patients in each age group. **b** the changes of the proportion of HFRS patients in each age group.

Table 2 presents the numbers and proportions of HFRS patients in each age group, stratified by year during the study period. The proportion of patients aged ≥70 years increased gradually from 16.4% in 2001 to 43.9% in 2017, whereas the proportion of patients aged 40–69 years decreased gradually from 60.1% in 2001 to 41.1% in 2017. Figure 1 demonstrates the gradual increases in both the number and proportion of HFRS patients aged ≥70 years since 2001.

**Table 2:** Number and proportion of HFRS patients according to age group in each year

Year	Total	Proportion of patients by age group		
		≤ 39 (n, %)	40~69 (n, %)	≥ 70 (n, %)
2001	323	76 (23.5)	194 (60.1)	53 (16.4)
2002	336	58 (17.3)	214 (63.7)	64 (19.0)
2003	392	84 (21.4)	245 (62.5)	63 (16.1)
2004	427	74 (17.3)	262 (61.4)	91 (21.3)
2005	421	70 (16.6)	255 (60.6)	96 (22.8)
2006	422	80 (19.0)	242 (57.3)	100 (23.7)
2007	450	66 (14.7)	266 (59.1)	118 (26.2)
2008	375	60 (16.0)	250 (66.7)	65 (17.3)
2009	334	57 (17.1)	182 (54.5)	95 (28.4)
2010	473	83 (17.5)	251 (53.1)	139 (29.4)
2011	370	55 (14.9)	195 (52.7)	120 (32.4)
2012	364	63 (17.3)	176 (48.4)	125 (34.3)
2013	527	102 (19.4)	255 (48.4)	170 (32.3)
2014	344	83 (24.1)	162 (47.1)	99 (28.8)
2015	384	82 (21.4)	168 (43.8)	134 (34.9)
2016	575	88 (15.3)	278 (48.3)	209 (36.3)
2017	531	80 (15.1)	218 (41.1)	233 (43.9)

Table 3 shows the proportions of patients in each age group according to quarter. The proportions of patients by quarter in the ≤39 years group were significantly higher in the first, second, and third quarters (15.7, 13.7, and 16.4%, respectively) and significantly lower in the fourth quarter (54.2%), compared to those in other age groups. By contrast, the proportions of patients by quarter in the ≥70 years group were significantly lower in the first and second quarters (6.9 and 8.9%, respectively) and significantly higher in the fourth quarter (69.6%), compared to those in other age groups. These data confirmed that the strongest seasonal variation was observed in the ≥70 years age group.

**Table 3:** Number and proportion of HFRS patients according to age group in each quarter

Age group	Quarters^a^			
	First quarter	Second quarter	Third quarter	Fourth quarter
Total (n, %)	719 (10.2)	725 (10.3)	952 (13.5)	4652 (66.0)
≤ 39 (n, %)	198 (15.7)^†^	173 (13.7)^†^	207 (16.4)^*^	683 (54.2)^†^
40~69 (n, %)	384 (10.1)	377 (9.9)	457 (12.0)^†^	2595 (68.1)^†^
≥ 70 (n, %)	137 (6.9)^†^	175 (8.9)^*^	288 (14.6)	1374 (69.6)^†^

All statistical significance was analyzed by χ^2^ test or Fisher’s exact test, appropriately

^a^The periods of each quarter are as follows: first quarter (January to March), second quarter (May to June), third quarter (July to September), and fourth quarter (October to December)

^*^P-value was less than 0.05, ^†^*P*-value was less than 0.001

**Fig. 2:**
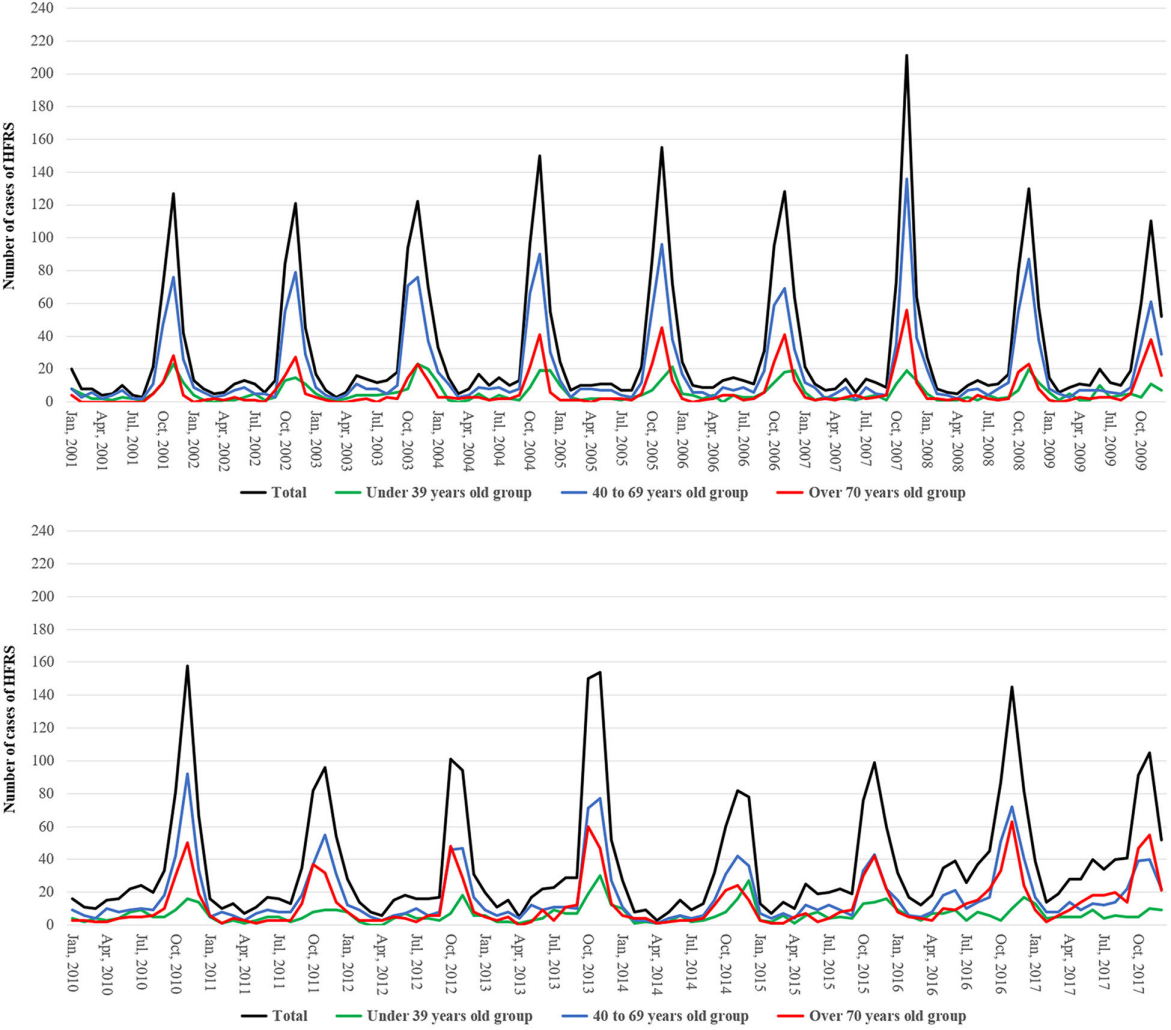
Monthly trends of the number of occurrence of HFRS for the past 17 years in each age group

Figure 2 shows continuous lines of HFRS occurrence in the past 17 years by overall and each age group. The monthly trends in HFRS incidence revealed that most cases occurred during the fourth quarter (October–December). Notably, the proportion of patients aged ≥70 years increased gradually throughout the study period, and this change corresponded with a distinct pattern of seasonal variation. Interestingly, in 2017, the number of patients aged ≥70 years was higher than the number aged 40–69 years.

Table 4 presents the results of a Poisson regression analysis of the RRs of time and sex with respect to HFRS incidence in each age group. The RRs for time were significantly higher among those aged ≤39 and ≥ 70 years old (1.021 and 1.076, respectively, vs. 0.979 for those aged 40–69 years), indicating that the greatest increase in HFRS incidence over time was observed among the oldest patients. The RR for sex was overall 1.631-fold higher for male relative to female sex. Notably, among patients aged ≤39 years, the RR for male sex was significantly higher than that in other age groups (4.820-fold).

**Table 4:** Poisson regression analysis for the effect of time and sex on HFRS incidence

	Age group	Relative risk (95% CI)	*P*-value
Time^a^	Total	1.002 (0.998–1.007)	0.348
	≤ 39	1.021 (1.010–1.033)	< 0.001
	40~69	0.979 (0.973–0.985)	< 0.001
	≥70	1.076 (1.066–1.086)	< 0.001
Male	Total	1.631 (1.555–1.711)	< 0.001
	≤ 39	4.820 (4.149–5.599)	< 0.001
	40~69	1.271 (1.192–1.355)	< 0.001
	≥70	1.543 (1.413–1.685)	< 0.001
Female	Total	Reference	
	≤ 39	Reference	
	40~69	Reference	
	≥70	Reference	

^a^As a continuous variable, the relative risk is due to an increase year

Dependent variable is the occurrence of HFRS

*CI* confidence interval

**Fig. 3:**
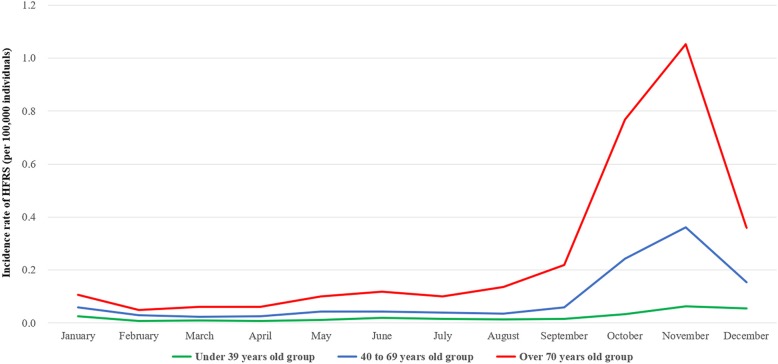
Monthly trends of the incidence rate of HFRS in each age group

Finally, the fourth quarter of the year was associated with significantly higher RRs for HFRS, with values of 6.470-fold overall and 3.449-, 6.758-, and 10.029-fold among patients aged ≤39, 40–69, and ≥ 70 years, respectively. In particular, the RRs for the third and fourth quarters among patients aged ≥70 years were 2.102 and 10.029, respectively, which were far higher than the corresponding RRs in the other age groups (Table 5). Among all age groups, those aged ≥70 years had the highest HFRS incidence per 100,000 individuals, exhibiting a distinct pattern of seasonal variation (Fig. 3).

**Table 5:** Poisson regression analysis for the effect of quarter on HFRS incidence

	Age group	Relative risk (95% CI)	*P*-value
First quarter^a^	Total	Reference	
	≤ 39	Reference	
	40~69	Reference	
	≥70	Reference	
Second quarter^a^	Total	1.008 (0.910–1.118)	0.875
	≤ 39	0.874 (0.713–1.071)	0.195
	40~69	0.982 (0.852–1.132)	0.800
	≥70	1.277 (1.021–1.597)	0.032
Third quarter^a^	Total	1.324 (1.202–1.459)	< 0.001
	≤ 39	1.045 (0.860–1.270)	0.655
	40~69	1.190 (1.039–1.363)	0.012
	≥70	2.102 (1.715–2.576)	< 0.001
Fourth quarter^a^	Total	6.470 (5.981–6.999)	< 0.001
	≤ 39	3.449 (2.945–4.041)	< 0.001
	40~69	6.758 (6.071–7.522)	< 0.001
	≥70	10.029 (8.414–11.954)	< 0.001

^a^The periods of each quarter are as follows: first quarter (January to March), second quarter (May to June), third quarter (July to September), and fourth quarter (October to December)

Dependent variable is the occurrence of HFRS

CI; confidence interval

## Discussion

This investigation of the epidemiology of HFRS in the ROK during the past 17 years revealed an average disease incidence per 100,000 individuals of 0.83 and a case fatality rate of 1.26%. More than half of all patients were male, and more than 80% were at least 40 years old. The present results were generally consistent with those of a previous study of HFRS epidemiology in the ROK from 2001 to 2010, which reported an incidence rate per 100,000 individuals of 0.81, a case fatality rate of 1.01%, a proportion of males of 57%, and a proportion of patients aged ≥40 years of 82.1% [11]. However, the previous study stratified patients into only two age groups, ≤39 vs. ≥40 years, whereas the present study added a third age group including patients aged ≥70 years.

Interestingly, the present study observed a gradual increase in the number and proportion of patients in this latter group, consistent with a previous study of 17 HFRS-endemic regions throughout China which showed that the proportion of patients aged ≥60 years increased gradually from 8.8% in 2005 to 14.7% in 2010 [15]. In China, the EPI was implemented in 2008 and targets the general population aged 16–60 years in HFRS-endemic regions. The authors observed that in these EPI-targeted regions, the proportion of patients aged 16–60 years decreased from 88.3% in 2007 to 81.6% in 2010, whereas that of patients aged ≥60 years increased from 9.2 to 14.7%. However, in non-EPI targeted regions, the proportion of patients aged ≥60 years also increased from 8.3% in 2007 to 10.3% in 2010. The authors of this Chinese study explained the reasons for this change in age distribution as follows. First, young people from rural areas migrate to urban areas, and the increased engagement of the remaining elderly population in work and increases in the average survival time lead to an increase in the percentage of elderly workers. Second, the EPI significantly reduced the disease incidence among patients aged 16–60 years, which led to a relative increase in the proportion of patients aged ≥60 years [15]. Another study conducted in Yichun City, China from 2005 to 2013 also observed a gradual increase in the proportion of HFRS patients aged ≥60 years in EPI-targeted regions, from 7.2% in 2005 to 21.6% in 2013, as well as a concomitant gradual decrease in the proportion of patients aged 16–60 years [23].

To date, hantavirus immunization initiatives in the ROK have not targeted specific age groups. However, the present study demonstrated gradual increases in both the number and proportion of HFRS patients aged ≥70 years during the past 17 years (Table 2, Fig. 1). Especially, a Poisson regression analysis revealed that the RR of time on the incidence of HFRS was highest among those aged ≥70 years compared to the other age groups (Table 4). This is probably attributable to overall population aging and a gradual decrease in the proportion of young people in rural areas of the ROK. Consequently, elderly residents are increasingly required to work and are more frequently at risk of exposure to hantaviruses; this is in line with the aforementioned explanation proposed by He et al. [15, 25].

In this study, a distinct pattern of seasonal variation in the incidence of HFRS was observed among patients aged ≥70 years (Fig. 2). Particularly, the Poisson regression analysis yielded an RR of 10.029 during the fourth quarter in this age group, which was significantly higher than the RRs of the other age groups (Table 5). Additionally, patients aged ≥70 years also had the highest incidence rate per 100,000 individuals (Fig. 3). Distinct patterns of seasonal variation in the occurrence of HFRS are usually attributable to HTNV infection, which primarily occurs in farming-related rural populations during the autumn harvest period [1, 26]. In contrast, the present study observed more similar proportions of patients aged ≤39 years across the four quarters of the year. In the ROK, this younger population includes soldiers who are predominantly male and perform many outdoor activities regardless of the season. In this study, 83.8% of patients aged ≤39 years were male, and the relatively lower level of seasonal variation in this age group was presumably due to the large number of soldiers. In other words, the increase in the HFRS incidence and the distinct pattern of seasonal variation among individuals aged ≥70 years are presumably due to an increase in the occurrence of HFRS among elderly agriculture workers in rural areas.

As noted previously, the lack of data on the effectiveness of the IHV from well-designed large-scale studies has hindered the active implementation of hantavirus immunization in the ROK [20, 21]. However, recently, a case-control study of Korean soldiers reported an adjusted hantavirus vaccine efficacy of 58.9%; the rate of 78.7% in the high-risk group suggests moderate effectiveness in this population [27]. In addition, He et al. and Liu et al. have reported in previous studies that immunization of high-risk group in general population is effective [15, 23]. Notably, the ROK also contains HFRS-endemic areas, such as Yeoncheon, Paju, and Cheorwon [11, 28, 29], and the present findings demonstrate an increased disease occurrence among elderly people in rural areas. Therefore, an immunization program that targets this high-risk population may be an important factor in controlling future occurrences of HFRS.

This study had some limitations, primarily related to the use of data reported to the KCDC. Specifically, information about the patients’ occupations was lacking, as these details are not necessarily provided when reporting a national notifiable infectious disease to the government of the ROK. Such data would have allowed a more selective determination of the population facing a high risk of HFRS. However, the patients’ occupations were indirectly estimated from factors such as seasonal variation patterns and sex. Still, this limitation is difficult to overcome.

## Conclusions

This study investigated the epidemiology of HFRS in the ROK over a relatively long time period and observed a gradual increase in the number and proportion of patients with HFRS aged ≥70 years. Additionally, this study detected a distinct pattern of seasonal variation among these older patients, indicating an increase in the incidence of HFRS among rural elderly populations. The controversy surrounding the effectiveness of hantavirus immunization has hindered its active implementation in the ROK. However, the IHV appears to be moderately effective in high-risk groups. In summary, this study provides important information regarding the selection of subpopulations at high risk of HFRS from the general population, which could be used to direct future efforts to control HFRS in the ROK. However, additional research will be needed to analyze the impact of IHV on the actual general population of the ROK.

## Abbreviations

EPI: Expanded Program on Immunization
HFRS: hemorrhagic fever with renal syndrome
HTNV: *Hantaan* virus
IHV: Inactivated hantavirus vaccine
KCDC: Korean Center for Disease Control and Prevention
NNDSS: National Notifiable Disease Surveillance System
ROK: Republic of Korea
RR: Relative risk
SEOV: *Seoul* virus
